# Human Papillomavirus Species-Specific Interaction with the Basement Membrane-Resident Non-Heparan Sulfate Receptor

**DOI:** 10.3390/v6124856

**Published:** 2014-12-05

**Authors:** Kathleen F. Richards, Santanu Mukherjee, Malgorzata Bienkowska-Haba, Jia Pang, Martin Sapp

**Affiliations:** Department of Microbiology and Immunology, Center for Molecular and Tumor Virology, Feist-Weiller Cancer Center, LSU Health Shreveport, Shreveport, LA 71130, USA; E-Mails: kfrichards1@gmail.com (K.F.R.); santanu69@gmail.com (S.M.); mbienk@lsuhsc.edu (M.B.-H.); gifanfang@hotmail.com (J.P.)

**Keywords:** HPV, extracellular matrix, heparan sulfate, laminin 332, receptor, entry

## Abstract

Using a cell culture model where virus is bound to the extracellular matrix (ECM) prior to cell surface binding, we determined that human papillomavirus type 16 (HPV16) utilizes ECM resident laminin (LN) 332 as an attachment receptor for infectious entry. In presence of LN332, soluble heparin can function as ligand activator rather than competitive inhibitor of HPV16 infection. We also show that the ability to use LN332 binding as a productive attachment step for infectious entry is not conserved amongst HPV types. In the alpha genus, species 9 members (HPV16) attach to ECM via LN332, while members of species 7 (HPV18) are completely inhibited by heparin pre-incubation due to an inability to use LN332. Since HPV species 7 and 9 are preferentially associated with adenocarcinoma and squamous cell carcinoma of the cervix, respectively, our data provide first evidence that pre-entry events may contribute to the anatomical-site preference of HPV species.

## 1. Introduction

Papillomaviruses (PV) is a family of non-enveloped, small DNA tumor viruses that have been found in all tested mammals and birds. PVs exhibit strict species specificity and the identification of viruses that cause lesions in multiple species is rare. There are over 120 types of human PV (HPV), which infect various sites throughout the human body and induce varying degrees of warts or papillomas [1]. HPVs preferentially infect either mucosal or cutaneous epithelium and replicate exclusively in terminally differentiating keratinocytes [2]. The cellular proliferation induced by HPV most commonly results in benign tumors and the viruses are eventually cleared by the host immune system. However, certain HPV types bear a higher risk for the development of lesions that are associated with the progression of a variety of malignancies [3,4]. Cervical cancer is the second most common cancer amongst women worldwide and shows complete association with “high-risk” HPV types, such as HPV16, HPV18, HPV31, HPV58, HPV45 and others [5,6]. The “high-risk” HPV types are also capable of causing other anogenital neoplasias and oral squamous cell carcinomas [7,8]. HPVs with low oncogenic potential (“low-risk”, such as HPV6 and HPV11) are associated with benign lesions known commonly as genital warts and are rarely found in high-grade lesions of the anogenital region [9]. The “high-” and “low-risk” HPV types are mucocutaneous and cluster in the alpha genus while cutaneous HPVs that are not associated with carcinoma in immunocompetent individuals are found mainly in the beta genus [1].

Classification of PVs is accomplished by comparison of the L1 major capsid protein gene, which is the most conserved gene within the genome [1]. In the PV virion, L1 is present in 360 copies and is organized into 72 pentamers, referred to as capsomeres [10,11,12]. These capsomeres assemble to form the outer virion shell and achieve a high degree of stability due to disulfide crosslinks between neighboring L1 molecules [12,13,14]. L2, the minor capsid protein, is not required to form a virus-like particle (VLP) and is predominantly hidden inside the L1 capsid shell of mature virions; however, it is necessary for the completion of infectious entry and increases the efficiency of encapsidation of the viral genome [15,16,17]. The PV virion encapsidates a circular chromatinized double-stranded DNA genome of approximately 8,000 bp. The inability of HPV to successfully propagate in non-differentiating cells has delayed discoveries of important steps in the attachment and entry process. However, the development of a pseudovirion (PsV) system that utilizes the natural ability of L1 and L2 to spontaneously assemble and encapsidate a reporter genome has overcome this obstacle. This PsV system has allowed for further investigation into the early events of the HPV infection process [18,19,20].

When considered in bulk as a population, attachment and entry of HPV is a slow, asynchronous process that involves multiple receptor engagements and conformational changes in both the L1 and L2 proteins, even though individual particles may be internalized quickly [21,22,23]. L1 mediates initial attachment to heparan sulfate (HS) proteoglycans (HSPG) present on the extracellular matrix (ECM) or cell surface of basal layer keratinocytes [24,25,26,27]. Sequential HS interactions with multiple HS binding sites on the viral capsid induce conformational changes in the capsid proteins, requiring the chaperone cyclophilin B (CyPB) and followed by cleavage of the L2 protein by furin convertase [28,29,30]. These conformational changes allow for transfer of the virus to a secondary non-HSPG receptor complex for uptake, endocytosis by a novel pathway termed micropinocytosis, and prepares the viral capsid for uncoating [23,31]. The secondary cell surface receptors involved in the uptake complex have not been fully elucidated. Currently, candidates include, integrins, tetraspanins, growth factor receptors and annexin A2 [32,33,34,35,36,37,38,39]. Following uncoating, cyclophilins facilitate dissociation of the L1 protein from the L2/DNA complex [40]. The L2/DNA complex traffics to the trans-Golgi network and requires mitotic progression, possibly in order to mediate nuclear envelope breakdown, for gaining access to the nucleus [41,42,43,44,45,46].

Beyond the species and cell-type restrictions, HPV types are repeatedly isolated from specific anatomical sites with little variability, suggesting some form of anatomical-site preference. In addition to the large groups of PVs showing tropism for mucosal versus cutaneous tissues, more subtle specificities have been observed. For example, HPV1 is mainly found in foot warts, whereas HPV2 is often associated with hand warts [9]. Similarly, HPV16 and HPV18 are the most common etiological agents of cervical cancer, yet squamous cell carcinoma is most commonly perpetrated by HPV16, while HPV18 infection accounts for a higher percentage of adenocarcinoma [3,47]. While reports have identified differential transcription regulation for certain HPV types, the mechanisms regulating anatomical-site preference amongst the high-risk species 7 and species 9 viruses have not been fully defined. Because the ubiquitous HS has been shown to serve as the attachment receptor *in vitro* and *in vivo* using keratinocyte-derived cell lines and the murine cervicovaginal infection model, respectively, receptor specificity is not considered to be responsible for tissue preference [48,49,50]. However, several groups have shown recently that the ECM resident laminin 332 (LN332, also known as LN5) can function as an attachment receptor for HPV6, HPV11, and HPV16 [51,52,53]. We have provided evidence that this interaction is of functional importance for successful HPV16 entry [28]. Others and we also demonstrated that in the presence of LN332 soluble heparin can increase rather than block infection of HPV16 pseudovirions, probably due to its ability to activate or induce the correct capsid conformations required for infectious entry [28,53]. Thus, in this context, heparin functions as a ligand activator of HPV16. We have now used an *in vitro* ECM to cell transfer assay to determine the ability of various HPV types to utilize the non-HS ECM receptor. By selectively inhibiting either HS or LN332, we provide evidence that LN332 functions as an HPV species-specific ECM-resident receptor, indicating that virus-receptor interactions may contribute to the anatomical-site specificity of HPVs. In addition, we propose that the LN332 binding event is necessary in order to allow virions, which are coated with soluble HS from the wound milieu, to specifically bind to the ECM. This model would account for the ability of PVs to avoid non-productive interactions with keratinocytes in the upper layers of the epithelium.

## 2. Materials and Methods

### 2.1. Cell Lines

HeLa and 293TT cells were cultured in Dulbecco’s modified Eagle’s medium (DMEM) supplemented with 10% fetal bovine serum (FBS), nonessential amino acids and antibiotics. HaCaT cells were grown in low-glucose DMEM containing 5% FBS and antibiotics. HTERT immortalized normal oral keratinocytes (NOK) cells were cultured in keratinocyte-serum free media supplemented with EGF 1–53 and Bovine pituitary extract. SVK cells were cultured in MEM Joklik modification containing 10% calf serum. Rona Scott, LSU Health Sciences Center Shreveport, LA, USA, provided both the SVK and NOK cell lines.

### 2.2. Plasmids and Pseudovirions (PsVs)

Plasmids containing codon-optimized L1 and L2 for all virus types (HPV16, 31, 33, 58, 18, 45, 6, 5, and BPV1) were used to transfect 293TT cells as described previously.(kindly provided by Martin Muller, German Cancer Research Center, Heidelberg, Germany and John Schiller, National Institutes of Health, Bethesda, MD, USA) [18]. PsV carrying a pEGFP (enhanced green fluorescent protein) reporter plasmid, where GFP expression is under the control of the SV40 promoter, were generated by simultaneous transfection of 293TT cells with L1/L2 plasmids and with the reporter plasmid. Particle yield was determined by green fluorescent protein cDNA-specific quantitative PCR. To control for variations during PsV generation, no less than 3 preparations were used for downstream analysis. Each PsV batch was separately quantified and analyzed to assure that experimental outcomes were reproducible between different PsV preparations. Every preparation underwent analysis for particle/DNA ratios, levels of L1/L2, and infection of 293TT cells, and only preparations with consistent results for all assays were used.

### 2.3. siRNA Knockdown of LN332

The three subunits of LN332 were knocked down using subunit-specific small interfering RNAs (siRNAs). The following *Silencer^®^* select siRNA from Ambion (Life Technologies, Grand Island, NY, USA) were used for knockdown of LN332; LAMA3-s8059, LAMB3-s8075, LAMC2-s8084. Knockdown was performed in HaCaT cells that were pre-incubated briefly with 0.5 mM EDTA in PBS. HaCaT cells were transfected with 6 µg total siRNA (2 µg/subunit) in a duplex with MATrA reagent (IBA Bio TAGnology, Goettingen, Germany), according to the manufacturer’s protocol. Twenty-four h post-transfection, cells were harvested and reseeded onto coverslips and 48 h later were processed for IF analysis. Knockdown of total LN332 was confirmed using western blot analysis with LN332 antibody detecting all three subunits (ab14509-Abcam).

### 2.4. Antibodies, Inhibitors and Reagents

Monoclonal antibodies 33L1-7 and H16.56E, which recognize a linear highly conserved epitope (residues 303-313 of HPV16 L1) and a conformational HPV16 L1-specific epitope, respectively, were described previously. Rabbit polyclonal HPV18 antibody raised against HPV18 VLPs was generously provided by Neil D. Christensen, Hershey Medical Center, Hershey, PA, USA [54,55,56]. A rabbit polyclonal antibody against LN332 (ab14509) (Abcam, Cambridge, MA, USA) was used to detect ECM depositions. The β-actin antibody was purchased from Santa Cruz Biotechnologies (sc-47778) (Dallas, TX, USA). Alexa Fluor-labeled secondary antibodies and Gold Antifade were purchased from Life Technologies. Heparin was purchased from Iduron (Manchester, UK). Marimastat (444289), batimastat (196440) and GM6001 (364206) were obtained from EMD Biochemicals (Darmstadt, Germany). For real-time PCR, SYBR-green from BioRad (Hercules, CA, USA) (IQ™ supermix^®^ #170-8880) was used. A Click-iT EdU imaging kit and Alexa Fluor-Labeled antibodies were purchased from Invitrogen. Peroxidase-conjugated AffiniPure goat anti-mouse antibodies were purchased from Jackson ImmunoResearch (West Grove, PA, USA). Synthesis of DSTP27, an *N,N'*-bisheteryl derivative of Dispirotripiperazine (DSTP) has been described before [57,58].

### 2.5. RNA Isolation and Quantification

Total RNA was isolated from HaCaT, 293TT, HeLa, NOK and SVK cells using TRIzol (Life Technologies, Grand Island, NY, USA). DNase-treated RNA was used to make cDNA using reagents purchased from Promega (Madison, WI, USA). For real time quantitative reverse transcription PCR (qRT-PCR), cDNA was amplified using oligonucleotides 5'-CTCACGGATGCGGGGTGCAC-3' and 5'-CCTGCGATGCCAGCTGGGTC-3' targeting LAMC2 (which encodes for the laminin γ2 subunit of LN332) as forward and reverse primers, respectively. Amplification of β-actin mRNA using oligonucleotides 5'-GGCATCCTCACCCTGAAGTA-3' and 5'-CAGAGGCGTACAGGGATAGC-3' as forward and reverse primers, respectively, was used for normalization. Negative controls included RT-negative samples and water replacing template.

### 2.6. ECM Cell Transfer Infection Assay

The indicated cells were seeded and allowed to grow for 48 h. Cells were then removed using 0.5 M EDTA (in PBS), leaving the ECM depositions on the growth surface. ECM was treated with DSTP27 (20 µg/mL), fixed, or left untreated for all experiments. After washing, PsV was added to the ECM depositions. When indicated, virus was pre-incubated with 10 µg/mL heparin for 30 min at 37 °C prior to addition to the ECM. Virus bound to the ECM was incubated for 1 h at 37 °C and then the wells were washed repeatedly with PBS. 293TT cells were used in all experiments as detector cells. Before addition, 293TT cells were either incubated with heparin (10 µg/mL), DSTP27 (20 µg/mL) or left untreated. Infection was scored 72 h post-addition of 293TT cells using flow cytometry. The MMP inhibition assay was performed by seeding HaCaT cells in the presence or absence of the BMG inhibitor cocktail and completing the infection assay as above. For all ECM to cell transfer assays, control wells lacking ECM were used to monitor infectious transfer of non-specific viral binding to the cell culture plate. The raw number of cells infected fluctuate experimentally for control infection and range from 1%–48%, however, all patterns of infection observed with various inhibitors and fixations stay constant. Graphs are the accumulation of percentage infections for at least 3 experimental replicates that each contains triplicates. For each HPV type, pseudovirions from at least 2 or more independent preparations were used for the transfer assays.

### 2.7. Enzyme-Linked Immunosorbent Assay (ELISA)

HaCaT cells were seeded into a 96 well plate for 48 h. Cells either were left untreated or were grown in the presence of a matrix metalloproteinase (MMP) inhibitor cocktail BMG (batimastat, marimastat and GM6001, all at a concentration of 20 µM). Cells were subsequently removed using 0.5 M EDTA (in PBS). PsV were diluted in PBS and added to the ECM-containing plate in replicates and allowed to bind for 1 h at 37 °C. Plates were then washed with PBST (PBS + 0.1%-Tween20). Wells were blocked using 0.01% bovine serum albumin (BSA) in PBST for 1 h at 37 °C. Primary antibodies were added for 1 h at 37 °C. Bound primary antibody was detected by the addition of a horseradish peroxidase (HRP)-coupled secondary antibody for 30 min at 37 °C. Assays were developed using trimethyl-benzidine (TMB, Promega, Madison, WI, USA) and stopped with 1 N HCL. Absorbance values were obtained at 450 nm using a FLUOstar-Omega plate reader (BMG Labtech, Ortenberg, Germany).

### 2.8. Immunofluorescence (IF)

**Figure 1:**
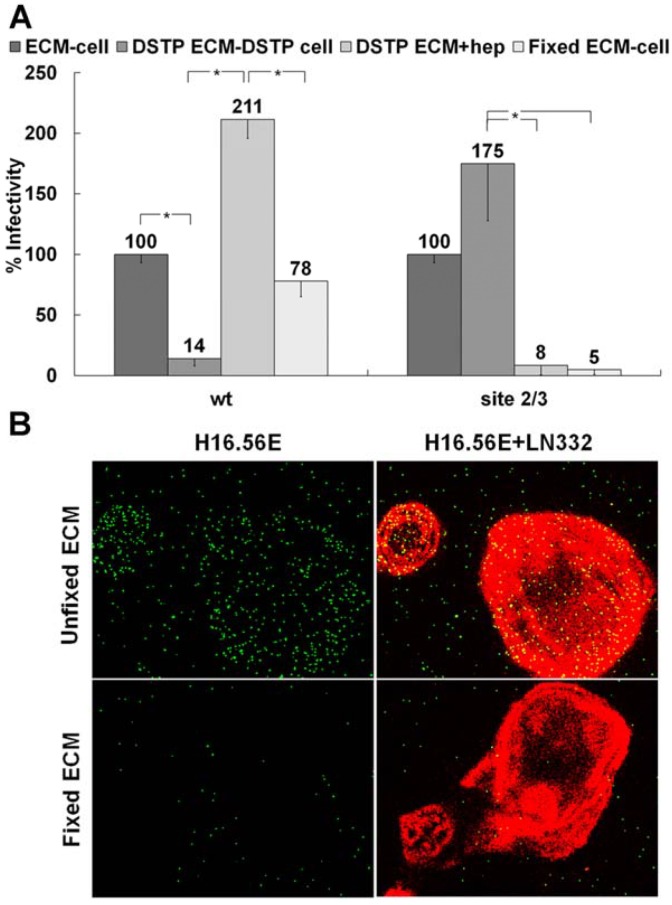
ECM cofactor facilitates infectious entry. (**A**) HPV16 wt or mutant site 2/3 (K54A/K356A-N57A/K59A-K442A/K443A) virus was allowed to bind to the ECM secreted by HaCaT cells. The ECM was untreated, treated with DSTP27 or fixed with 4% PFA. When indicated, 293TT cells were also treated with DSTP27. For the DSTP ECM+hep, detector 293TT cells were incubated with 10 μg/mL heparin. Infectious transfer to 293TT cells was scored using flow cytometry 72 hpi; (**B**) HaCaT cells were grown on coverslips for 48 h and subsequently removed with 0.5 M EDTA in PBS. The ECM depositions were either untreated or fixed with 4% PFA. Wt HPV16 virus was incubated with 10 µg/mL heparin and then added to coverslips. LN332 reactivity is shown in red; virus was detected with H16.56E (added prior to fixation of the virus) and is shown in green. When the statistical significance of a data set as determined by a Student’s *t*-test differed from control by *p* ≤ 0.001, it is indicated in the graph (*).

**Figure 2:**
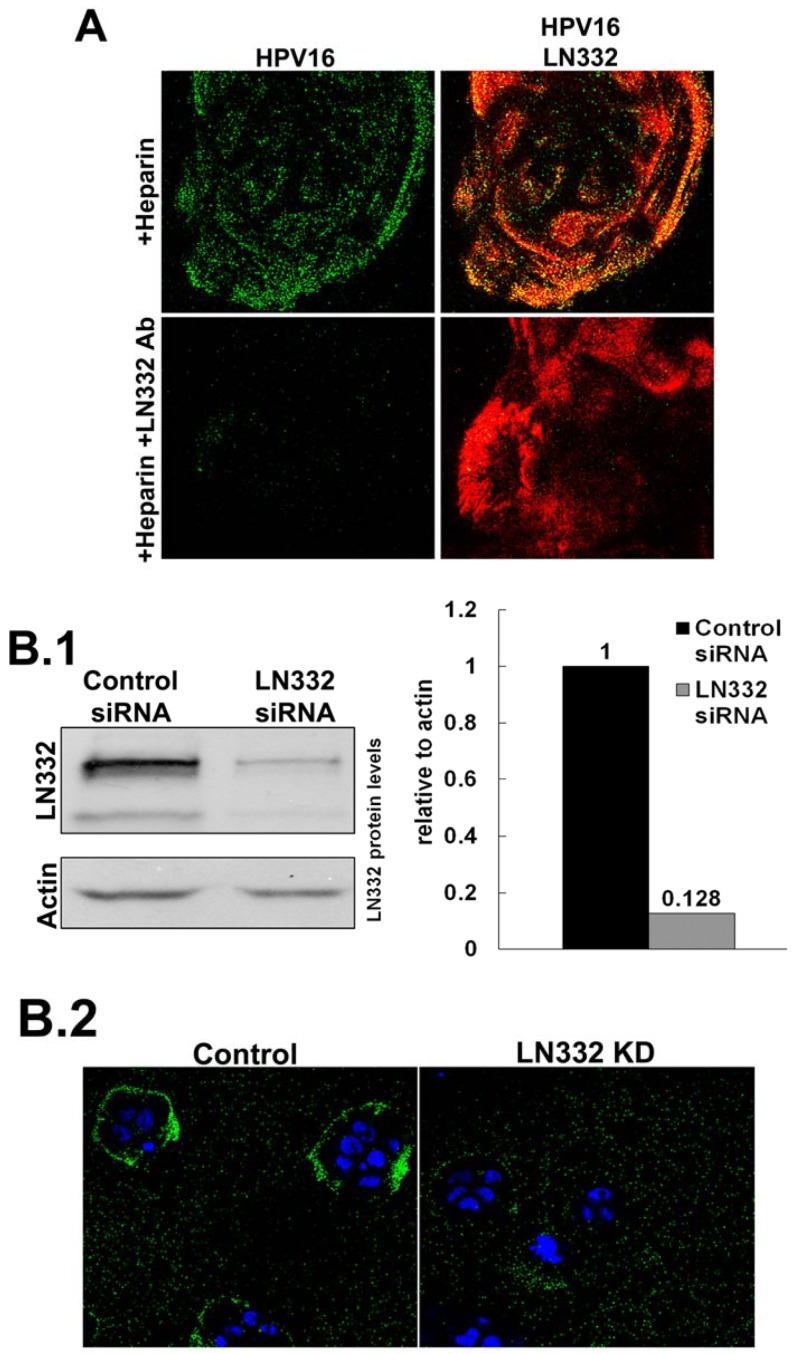

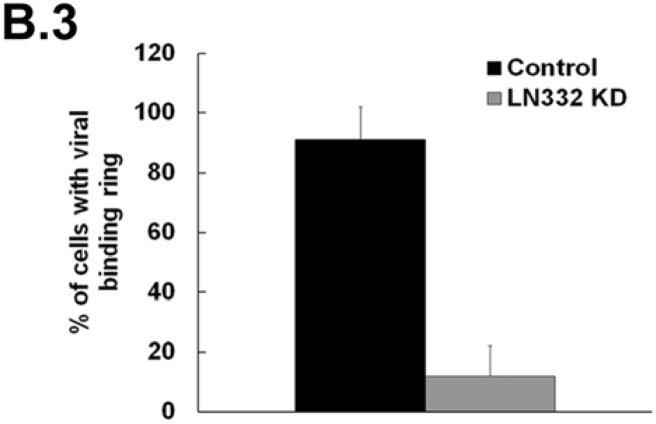
HPV16 utilizes LN332 to bind to the ECM in the presence of heparin. (**A**) Virus was allowed to bind to the ECM in the presence of heparin (10 g/mL). The ECM was either untreated or treated with LN332 polyclonal antibody prior to viral binding. Virus (green) was detected using 33L1-7, after denaturation, and ECM (red) was detected with LN332 antibody; (**B**) LN332 subunits were knocked down using siRNA in HaCaT cells; (**B.1**) Western blot for LN332 and Actin in control and siRNA treated cells. Protein levels were determined from blot image using densitometry with actin as a loading control; (**B.2**) Wt HPV16 virus was incubated in the presence of heparin at a 10 μg/mL, added to control and LN332 KD HaCaT cells. Cell nuclei are stained with DAPI (blue) and virus is detected with 33L1-7 (green); (**B.3**) The proportion of cells possessing a ring of virus bound to the secreted ECM of control and LN332 KD HaCaT cells were counted.

HaCaT cells were seeded into 6 well dishes and allowed to reach approximately 50% confluence. For the measurement of ECM binding in the absence of cells, 0.5 M EDTA (in PBS) was used to remove the HaCaT cells. Virus was added to either HaCaT cells or HaCaT cell ECM depositions. Where indicated, virus was pre-incubated with 10 µg/mL of heparin prior to addition to the cells/ECM. Virus was allowed to bind for 1 h at 37 °C, followed by extensive washing. In Figure 1 and Figure 2A H16.56E was added prior to fixation for 1 h at 37 °C, followed by washing and fixation with 4% paraformaldehyde (PFA). For staining of siRNA knockdown slides (Figure 2B), 33L1-7 was used to detect virus. In all cases, anti-LN332 antibody was used to detect ECM depositions. For the LN332 blocking experiment (Figure 2A), a high concentration of LN332 antibody (1:25 dilution) was added to the ECM prior to viral binding followed by multiple PBS washes. After addition of fluorescently labeled secondary antibodies, slides were mounted onto coverslips using Gold Antifade, which contains 4',6-diamidina-2-phenylindole (DAPI) for detecting nuclei. For detection of virus with 33L1-7 in Figure 2B viral particles were denatured as described previously using the Click-iT reaction cocktail (without the addition of the EdU label to virions or subsequent Alexa Fluor staining) [40]. In Figure 4, different HPV types were detected with the following antibodies: HPV16-33L1-7, HPV18-33L1-7, HPV45-33L1-7, HPV31-33L1-IID5, 16-312F and 33L1-7. In this experiment coverslips were incubated with warm (37 °C) 0.2% SDS in order to expose antibody binding sites prior to fixation. All immunofluorescence (IF) images were captured by confocal microscopy with a 63X objective (Leica TCS SP5 spectral confocal microscope) and processed minimally with Adobe Photoshop (CS4).

## 3. Results

### 3.1. Presence of an ECM Receptor that Supports Infectious Transfer of HPV from the ECM to the Cell Surface

We have recently reported our findings on multiple HS binding site-deficient HPV16 pseudoviruses, which were generated by mutating the identified HS interaction sites on the viral capsid. These pseudoviruses are defective for infection if directly bound to the cell surface [28]. We determined HS binding site mutant viruses with intact primary binding site but mutated secondary binding sites could gain infectivity in an ECM cell transfer assay. In this assay, HPV16 pseudovirions are pre-bound to the ECM secreted by HaCaT keratinocytes prior to the addition of indicator cell lines, such as 293TT, HeLa or HaCaT cells [52]. This allows individual manipulation of ECM depositions and cell surface interactions. Figure 1A confirms our previous findings [28]. Whereas blockage of ECM-resident HS using DSTP27, which binds to HS preventing viral binding [52,57,58], reduces infectious transfer to 293TT cells, addition of heparin together with indicator cells restores infection. If DSTP27 is added to the ECM and cells prior to virus attachment and cell surface transfer, respectively, wt pseudovirus shows decreased infectivity due to the infection being dependent on HS engagements. In contrast to wt pseudovirus, a mutant pseudovirus that is lacking all known secondary HS binding sites referred to as site 2/3 (K54A/K356A-N57A/K59A-K442A/K443A) shows enhanced infectivity as availability of HS interactions is decreased (Figure 1A). The levels of infection for the mutant pseudovirus in the presence of heparin are reduced to the level of infection without ECM pre-binding, indicating this virus only successfully completes infection if allowed to bind in an HS-independent manner to the ECM prior to the addition of 293TT cells. These findings provide us with the means to separate the functions of the non-HS ECM receptor and HS during infectious entry. We next tested whether the ECM receptor, which is responsible for viral binding in the absence of HS (DSTP27 treatment), is sensitive to fixation. If the ECM was fixed with 4% PFA prior to viral binding, wt infection was not significantly affected. However, the mutant pseudovirus is rendered non-infectious (Figure 1A). Similar to previous reports, these data indicate there is a non-HS receptor present in the ECM for HPV16 that is sensitive to fixation and is capable of supporting infectious transfer of virions to the cell surface.

We corroborated our findings by immunofluorescence using heparin as a competitive inhibitor of binding to HS. Heparin has been shown previously to prevent cell surface binding and allows for direct analysis of ECM binding [59]. Soluble heparin does not block binding of HPV16 PsV to the ECM. Following fixation with PFA, however, exogenous heparin was able to completely prevent binding of HPV16 particles to ECM (Figure 1B).

### 3.2. LN332 is Required for Infectious Transfer of HPV16 in the Presence of Heparin

Previously published data indicate a role for LN332 in the infectious entry of HPV [51,53,60]. To test this directly, we performed siRNA knockdown of all three subunits of LN332 in HaCaT cells (Figure 2B). Successful knockdown was confirmed by Western blot analysis. When LN332 is present in the ECM, HPV16 is capable of binding to the ECM rings that are secreted from HaCaT cells in the presence of heparin. When LN332 is knocked down, this binding is diminished. These data confirm that LN332 is the non-HS ECM receptor for HPV16 and is required for binding to the ECM when virus is first incubated with heparin. The knockdown efficiency was not good enough to significantly alter the infectivity of HPV16 in the ECM to cell transfer assays probably because this assay is much more sensitive than the IF analysis (data not shown). However, in line with our previous findings, LN332 antibodies not only blocked binding (Figure 2A; [52]) but also infection in ECM to cell transfer assays in presence of DSTP27 [52].

### 3.3. Investigating the Ability of Multiple HPV Types to Use a Non-HS Receptor in the ECM

**Figure 3:**
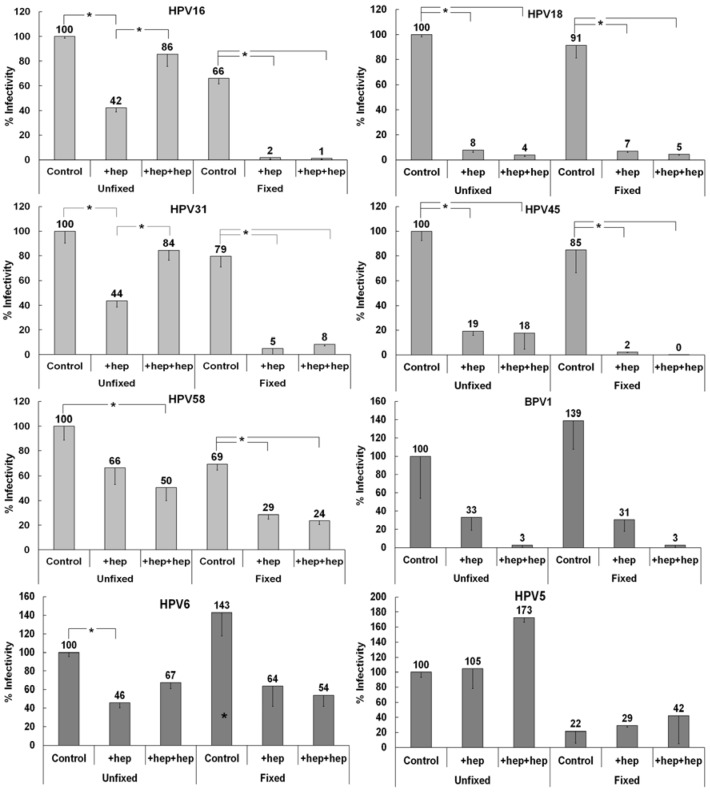
Infectivity patterns of various HPV types in the ECM to cell transfer assay. Viruses were bound to either control or fixed HaCaT ECM. Heparin was added to 10 µg/mL either with the virus (+hep) or with both the virus and after significant washing, the indicator 293TT cells (+hep+hep). Infectivity of each virus type was normalized to infectivity for control ECM to cell transfer of that virus type. When the statistical significance, as determined by a Student’s *t*-test, of a data set differing from the control with *p* ≤ 0.001, it is indicated in the graph (*). The (*) above the control infection on fixed ECM for HPV5 indicates that this was significant compared to control infection on unfixed ECM.

To explore non-HS ECM receptor usage by different HPV types, we developed an ECM to cell transfer assay that would more closely mimic the *in vivo* scenario of heparin availability versus DSTP27 treatment. HPV must access the extracellular matrix surrounding basal layer keratinocytes in order to bind and eventually establish successful infection [22,49]. In the normal wound-healing environment that would be induced by the epithelial trauma event that allows for entry of HPV, there is an abundance of heparan sulfate [61,62]. In our ECM to cell transfer assay, we can more easily duplicate this situation using heparin incubation with virus prior to ECM binding and incubation with detector cells. For this assay, we allowed virus to bind either fixed or unfixed ECM, in the presence or absence of heparin, and then added indicator 293TT cells, also with or without heparin. The addition of heparin during ECM binding should allow for the targeting of the virus to the non-HS ECM receptor, while addition of heparin with the detector cells further inhibits interactions with cell surface HSPG and limits binding to the non-HS receptor(s) on the cell surface. We used HPV16 to develop a baseline for ECM receptor usage since we have studied this type in depth previously with DSTP27 [52]. Similar to the DSTP27 experiments, HPV16 is inhibited by heparin added during ECM binding, but infectivity was restored when heparin was also added with detector cells (Figure 3). If the ECM was fixed prior to viral binding, the only successful infection was in the absence of heparin.

We first tested multiple “high-risk” types of HPV for their ability to use a non-HS ECM receptor in a similar manner to HPV16 using pseudovirions. Pseudoviruses that belong to the same species as HPV16 (species 9) showed similar ECM to cell transfer patterns (Figure 3). HPV31 is inhibited by heparin addition, although addition also with detector cells restored infectivity close to the control level. This interaction, similar to that of HPV16, is inhibited by fixation of the ECM. HPV58 is neither fully inhibited by heparin, nor does it show increased infectivity when heparin is added with detector cells. However, HPV58 is still inhibited by the addition of heparin following ECM fixation. 

We next tested HPV18 and HPV45 (species 7) and found that both of these virus types have a distinct pattern of non-HS ECM receptor usage. Heparin addition uniformly causes decreased infectivity, whereas fixation alone has negligible effects. This indicates an inability of these viruses to use the non-HS ECM receptor that viruses of species 9 use for infectious transfer in the presence of heparin.

BPV1, a member of species 4 in the delta papillomavirus genus, shows decreased infectivity in the presence of heparin independent of fixation (Figure 3). This pattern of non-HS receptor usage is similar to those viruses in species 7, indicative of an inability to use the non-HS receptor present in the ECM for infectious transfer.

We further investigated differential non-HS ECM receptor usage by testing members of various groups and species. HPV6, a “low-risk” member (species 10) of the alpha papillomavirus genus, displays an intermediate phenotype (Figure 3). Infectivity is inhibited by early heparin addition and is only slightly increased when heparin was added with detector cells as well. Distinct from previous HPV types tested, HPV6 that is pre-incubated with heparin is not strongly affected by fixation of the ECM. HPV5, a member of species 1 in the beta papillomavirus genus, is unaffected by the addition of heparin and shows enhanced infectivity when heparin is added also with detector cells (Figure 3). HPV5 shows a strict reliance on the non-HS ECM receptor and displays the lowest infectivity in all cases when the ECM was fixed prior to binding. Taken together, these results are consistent with the idea that different HPV types use an ECM receptor to varying degrees in order to complete infectious entry.

### 3.4. Monitoring ECM Binding Patterns for Different HPV Types

**Figure 4:**
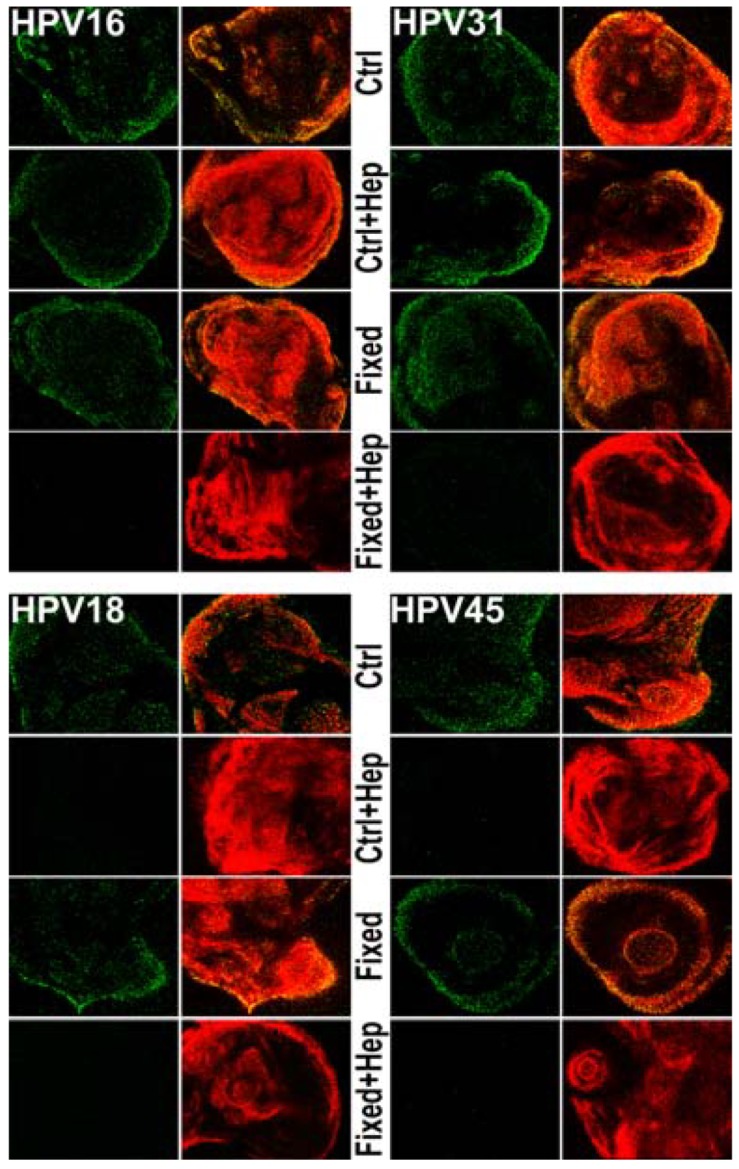
Monitoring viral binding to the ECM in the presence of heparin. Virus was allowed to bind to HaCaT ECM for 1 h at 37 °C. Following fixation, capsids were denatured using a short SDS wash. Virus (green) was then stained with 33L1-7 or a pool of monoclonal antibodies and the ECM (red) was detected using LN332 rabbit polyclonal antibody. Where indicated, the ECM was fixed with 4% PFA prior to viral binding. Heparin (10 µg/mL) was incubated with viral particles before the ECM binding reaction, where indicated.

To determine if the mechanism by which heparin blocks the infectious transfer of HPV18 and HPV45 is at the ECM binding step of infection, we monitored ECM binding using immunofluorescence (Figure 4). HaCaT ECM was generated on coverslips and was left unfixed or fixed with 4% PFA. Viral particles were untreated or incubated with 10 µg/mL heparin prior to binding. LN332 antibody was used to detect the ECM. HPV16, HPV45 and HPV18 were detected using 33L1-7 following short denaturation in SDS. HPV31 was detected with a pool of monoclonal antibodies after denaturation. All virus types bind to the ECM whether it is fixed or unfixed. However, when virus is incubated with heparin prior to binding, HPV18 and HPV45 were no longer capable of binding to control ECM. In contrast, HPV16 and HPV31 bound to unfixed ECM in the absence or presence of heparin, whereas both failed to bind when heparin was present and the ECM was fixed, which presumably destroys the LN332 epitope used for viral binding. The inability of HPV18 and HPV45 to bind to the ECM under any conditions in which heparin is present indicates a reliance of viral particles on HS for all steps of infection prior to interaction with the uptake receptor. These results directly correlate to the inability of species 7 viruses to use the non-HS ECM receptor for infectious transfer.

### 3.5. Determining if the Non-HS ECM Receptor is Found in the Secreted ECM of Various Keratinocytes

**Figure 5:**
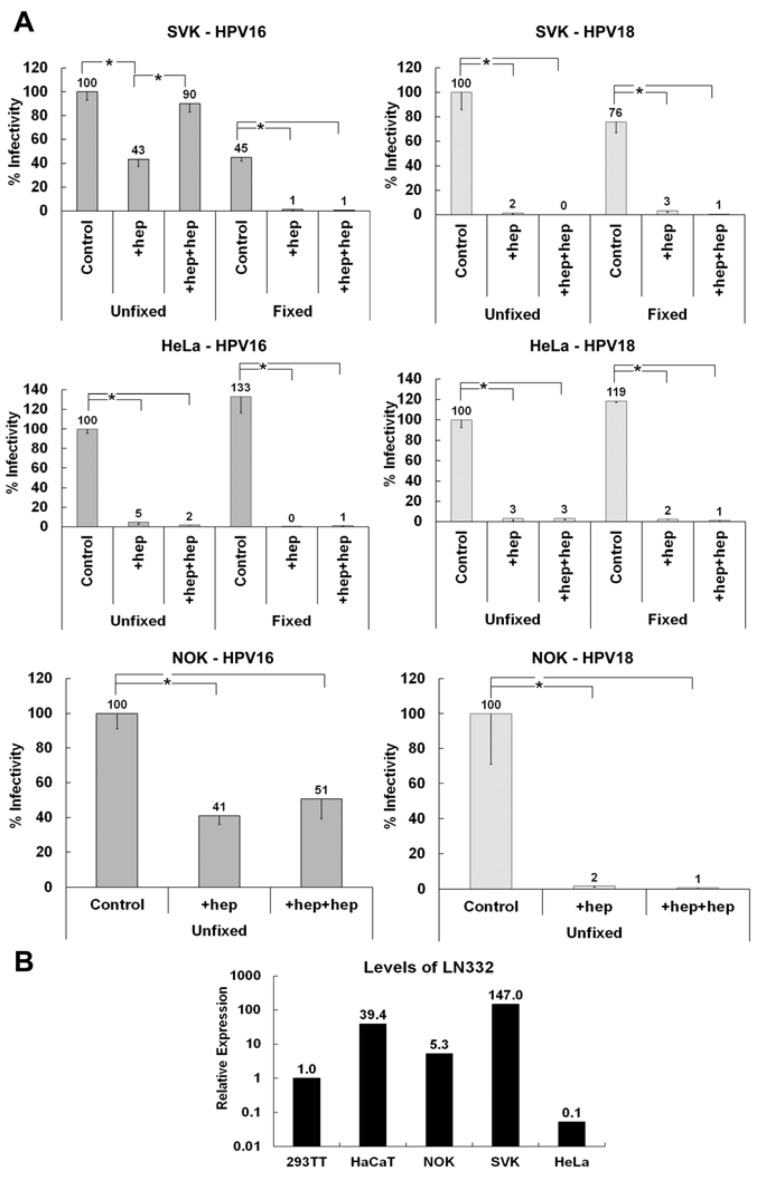
Investigating the ability of HPV16 and HPV18 to utilize the ECM of various cell lines for infectious transfer. (**A**) HPV16 and HPV18 viruses were allowed to bind to the ECM secreted by SVK (SV40 transformed keratinocytes), NOK (normal oral keratinocytes) or HeLa cells in the presence or absence heparin at a 10 μg/mL. Indicator 293TT cells were added and infection was scored 72 hpi; (**B**) Expression levels of LAMC2 (LN332) were determined using qRT-PCR on total RNA isolated from indicated cells. All graphs show average values from at least three experiments containing triplicates. When the statistical significance of a data set as determined by a Student’s *t*-test differed from control by *p* ≤ 0.001, it is indicated in the graph (*).

Our data suggest that HPV16 but not HPV18 utilizes the non-HS ECM receptor LN332 for infectious transfer. To confirm these findings and in a search for a non-HSPG receptor for HPV18, we tested the secreted ECM of normal oral keratinocytes (NOK) and SV40-transformed keratinocytes (SVK) in the ECM to cell transfer assay using pseudovirions. The ECM of both cell lines supports infectious transfer of HPV16 when heparin is added before binding and with detector cells (Figure 5A). However, these cell lines are unable to support the infectious transfer of HPV18 in the presence of heparin. Interestingly, when the ECM of HeLa cells are used in the assay, neither HPV16 nor HPV18 infectiously transferred to the cell surface if heparin is present during any step of infection. To confirm the expression of LN332 in NOK and SVK but not HeLa and 293TT cells, we performed quantitative reverse transcriptase PCR using RNA isolated from these cells. We found that NOK and SVK but not HeLa and 293TT cells expressed appreciable levels of LN332 (Figure 5B). This result further supports the idea that LN332 acts as a non-HS ECM receptor that allows HPV16 but not HPV18 ECM to cell transfer even in the presence of heparin.

### 3.6. Matrix Metalloproteinase Processing of LN332 Does Not Affect HPV18 Dependence on HS

**Figure 6:**
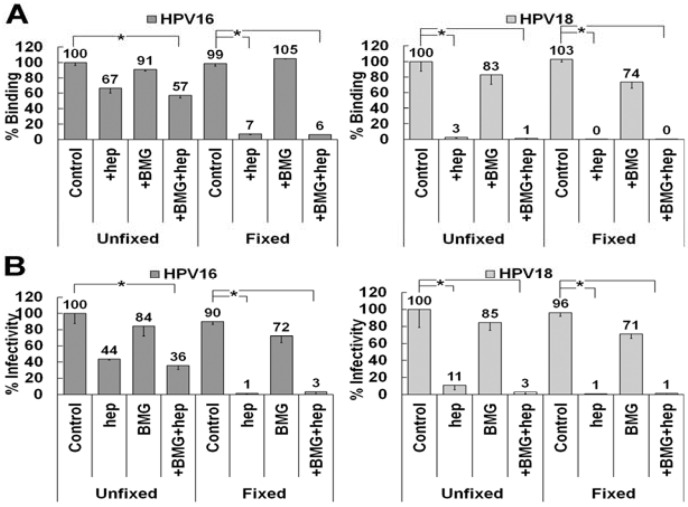
LN332 processing by matrix metalloproteinases (MMPs) does not affect ECM to cell transfer of HPV16 or HPV18. (**A**) HPV16 and HPV18 virus preparations were allowed to bind control or fixed ECM of HaCaT cells in the presence or absence of 10 μg/mL heparin. HaCaT were treated with MMP inhibitors (BMG) prior to removal. Virus was allowed to bind for 1 h and then detected using HPV16 or HPV18 antibodies in an ELISA assay. Absorbance was determined at 450 nm and binding in expressed is a percentage of control binding for both virus types; (**B**) HPV16 and HPV18 virus was allowed to bind to control or fixed ECM of HaCaT cells in the presence or absence of 10 μg/mL heparin. The HaCaT were treated with MMP inhibitors (BMG) prior to removal. 293TT indicator cells were added with or without 10 μg/mL heparin and infectivity was scored 72 h later. When the statistical significance of a data set as determined by a Student’s *t*-test differed from control by *p* ≤ 0.001, it is indicated in the graph (*).

Our data show that HPV16 can use LN332 as an ECM attachment factor in the presence of heparin on the ECM secreted by HaCaT cells, whereas HPV18 cannot. LN332 undergoes various amounts of processing in the ECM. We hypothesized that HPV18 may be able to use LN332, but only under circumstances where the protein has not been enzymatically processed by matrix metalloproteinases (MMPs) [63,64,65]. To test this possibility, we inhibited the activity of MMPs using a broadly active mixture of inhibitors at concentrations previously shown to block protease activity and tested the ability of HPV16 and HPV18 to bind to the ECM in the presence of heparin (Figure 6A) [66,67,68]. MMP inhibition had no effect on HPV16 binding to fixed or unfixed ECM in the presence or absence of heparin. HPV18 did not utilize a non-HS ECM receptor in ECM to cell transfer assays whether or not LN332 processing was inhibited (Figure 6B). These data indicate that MMP processing is not responsible for the lack of HPV18 binding to the ECM in the presence of heparin. Thus we conclude that HPV18 either has a completely different receptor molecule or no non-HS ECM receptor at all.

## 4. Discussion

**Figure 7:**
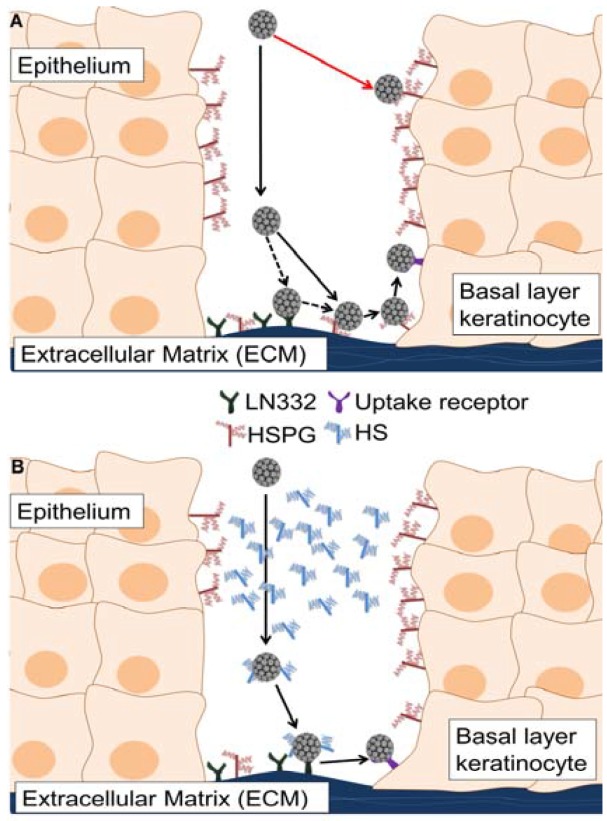
A model of HPV16 entry in the presence of soluble heparan sulfates. Infection with HPV occurs following a wounding event, which allows viral particles access to the basal layer of the epithelium. If the wound exudate is not taken into account, HPV infection would proceed as in the top panel (**A**); HPV would undergo unproductive interactions with suprabasal cell surface heparan sulfate (HS) and virus transit to the extracellular matrix (ECM) would be inefficient. Virus would bind to HS or LN332 present in the ECM, transfer to cell surface HS, interact with the non-HS uptake receptor and then be infectiously internalized after completing all necessary conformational changes. If the wound-healing environment is considered, a more appropriate model of entry is shown in the bottom panel (**B**). In this scenario, which we used the *in vitro* ECM to cell transfer assay to mimic, virus would encounter HS in the upper epithelial layers and be targeted to the ECM. The HS-coated particle binds to LN332 and, following HS-dependent conformational changes, is prepared for infectious entry into wound keratinocytes. In (**B**) soluble HS and LN332 substitute for ECM- and cell surface-resident HS.

While most viruses initiate infection by binding to the cell surface, PV may have evolved to interact initially with ECM-resident receptors. HPV binding to the extracellular matrix *in vivo* has been described as a binding event that occurs prior to interaction with the cell surface [22]. *In vitro*, preferential viral binding to the ECM has been observed, although this interaction is not critical for infectious entry and virus is capable of infecting cells in the absence of a substantial secreted matrix (293TT cells). The completion of the PV life cycle requires initial infection of basal layer cells that are in contact with the ECM. During natural infections, virions likely encounter HS present on the surface of differentiated keratinocytes and as soluble molecules secreted by wound healing keratinocytes. Based on data presented herein, we propose that HPV bypasses unproductive interactions with cell surface HS in the upper layers of the epithelium by forming complexes with soluble HS. The HS-coated virus will thus be directed to bind to the non-HS ECM receptor, LN332 in the case of HPV16, allowing for transfer to the basal cell keratinocytes and establishment of a productive infection (Figure 7).

Our laboratory’s recent publication highlights the requirement for HS interactions *in vitro* and introduces a model for attachment and entry that relies on ECM binding and multiple HS engagements [28]. Multiple HS interactions are essential for required conformational changes to occur, thus allowing transfer to uptake receptors and uncoating following internalization. In the presence of LN332, however, soluble HS rather than ECM or cell surface HS can function as a ligand activator for HPV16 by binding to and inducing conformational changes of both capsid proteins if necessary host cell factors, such as CyPB and furin convertases, are present. The relevance of this non-HS binding partner in the ECM to productive infection can be seen most clearly when using the L1-K54A/K356A-N57A/K59A-K442A/K443A mutant virus, which lacks all secondary HS binding sites. This mutant virus becomes fully infectious in the absence of ECM and cell surface resident HS if LN332 is functional (*i.e.*, the ECM is not fixed prior to viral binding). HPV16 wt binding to the ECM in the presence of heparin is restricted to depositions containing LN332, as determined by antibody blocking and knockdown experiments, again confirming a key role for this receptor. These data allow us to hypothesize that LN332 may act as an *in vivo* receptor for HPV16 that mediates targeting of the virus to the interface between the ECM and basal layer keratinocytes even in the presence of excess soluble HS.

The ability to selectively inactivate the two ECM-resident HPV16 receptors allowed us to examine the relevance of LN332 for binding and infection of PV types grouped into different species and genera. We began by testing “high-risk” HPV types and found infectivity patterns that were relatively similar amongst types of the same viral species. Members of species 9, including HPV16, HPV31 and HPV58, are capable of completing infectious ECM to cell transfer in the presence of heparin by binding to a non-HSPG receptor. These results are in striking contrast to what is seen when HPV18, HPV45 and BPV1 are tested in this assay, which indicates the lack of an appropriate non-HS ECM molecule for these virus types in the HaCaT cell secretions. These results are mirrored when monitoring ECM binding by IF and confirm that the distinct difference in infection patterns is the product of the ability of viral particles to bind to the ECM in the presence of heparin. Another member of the alpha papillomavirus family, HPV6, can use the non-HS ECM receptor even in the presence of fixation. HPV6 and HPV11 have previously been shown to bind LN332 by the Christensen lab [51,69]. Our data may indicate that there is another receptor in the ECM capable of supporting HPV6 or, alternatively, that HPV6 and HPV16 use different domains of LN332 with different sensitivities to fixation. We tested one member of the beta papillomavirus family, HPV5; this virus is unaffected or enhanced by the presence of heparin and is incapable of infectious transfer when the ECM is fixed, however, is sensitive to de-N-sulfated heparin [49].

To further investigate the difference in ECM receptor usage between HPV16 and HPV18, we tested various cell lines for their ability to secrete the appropriate non-HS receptor for HPV16 and also to determine if another cell type could support the ECM to cell transfer of HPV18 in the presence of heparin. We report here that NOK and SVK cells express and secrete the non-HS ECM receptor for HPV16 but not HPV18. Interestingly, HeLa cells do not support infectious transfer in the presence of heparin for either HPV16 or HPV18. These cells are commonly used in cell culture to study HPV infection and are clearly able to be infected by these types of HPV if virus is allowed to bind to the cell surface directly or in the absence of heparin. In contrast to HaCaT, NOK and SVK cells, HeLa cells do not secrete LN332 into the ECM and this coincides nicely with our and other’s data indicating involvement of LN332 during HPV16 infection [52,53]. Accordingly, knockdown of the three subunits of LN332 in HaCaT cells prevents HPV16 binding to the ECM in the presence of heparin. Our inability to monitor ECM to cell transfer in the LN332 knockdown cells is likely due to the presence of LN332-rich ECM depositions that attract high levels of heparin-coated viral particles. However, we provide evidence that ECM binding ability consistently correlates with the outcome of infection. Similarly, infection in the absence of human LN332, such as in HeLa cells or mouse keratinocytes, is inhibited by heparin. These data provide substantial evidence that LN332 is a participant in the infectious entry of HPV16 [52,53].

HPV species exhibit anatomical site infection preference in that they induce epithelial lesions at different anatomical regions. HPV16 and HPV18 as representatives of species 9 and 7, respectively, are the most commonly found HPV types in cervical cancer. However, species 9 and 7 also display some anatomical-site preference as they are more commonly found in squamous cell carcinoma and adenocarcinoma, respectively. Along with other risk factors, squamous cell carcinoma occurs due to high-risk HPV infection of the squamous epithelium in the surface lining of the ectocervix [70,71]. Conversely, high-risk HPV infection-induced adenocarcinoma arises from the glandular cells located in the endocervix [72]. Despite the close proximity of these regions in the cervical anatomy, the infection of a distinct anatomical site contributes to the tumorigenesis of a high percentage of HPV16- and HPV18-induced carcinomas [47,73,74]. It is interesting to speculate that the difference for distinct cellular tropism may be a result of the availability of specific extracellular factors, such as LN332. While we have determined that keratinocytes in culture secrete LN332, which HPV16 can use for ECM to cell transfer, so far we don’t have evidence that HPV18 is able to utilize any molecule other than HS to mediate infectious transfer. Because LN332 is subject to proteolytic processing by MMP and ADAM proteases, we used highly promiscuous inhibitors of MMPs and ADAM proteases to prevent putative processing from taking place. We do not see a gain in HS-independent binding or infectivity of HPV18 with ECM lacking MMP processing, indicating that the inability of HPV18 to infectiously transfer to the cell surface in the presence of heparin is not due to proteolytic processing of LN332 that can be blocked by these inhibitors. These data leave us with multiple hypotheses that will require further investigation: either HPV18 uses a distinct form of LN332 that is not processed by MMPs or produced by the cell lines we have tested, is able to bind to a non-HS ECM receptor other than LN332, or does not use separate attachment and entry factors other than HS and the non-HS uptake receptor complex. We want to stress again that the non-HS ECM receptor is not absolutely necessary but may rather favor infection of cells expressing the appropriate non-HS receptor.

In summary, our data highlight the ability of HPV16 and related viruses to utilize a non-HS ECM receptor, LN332. This binding may serve to facilitate attachment of virions that have encountered HS in the upper layers of the epithelium and can no longer bind to cell surface- or ECM-resident HSPGs. While the idea of extracellular regulators of viral tropism has been explored previously, many of these studies failed to take ECM receptors into account [49,75]. Our study provides evidence that infection that starts with ECM binding is distinct from the most common *in vitro* approach of infecting pre-plated cells. It also points to the possible importance of pre-entry events in viral tropism and thus challenges the widely held beliefs that post entry events are exclusively responsible for the observed anatomical-site preferences.
